# The complete chloroplast genome sequence of *Tamarix arceuthoides* Bunge and *Tamarix ramosissima* Ledeb. (Tamaricaceae)

**DOI:** 10.1080/23802359.2023.2209215

**Published:** 2023-05-11

**Authors:** Xiyong Wang, Qiumei Cao, Yan Wei

**Affiliations:** a College of Grassland Sciences, Xinjiang Agricultural University, Urumqi, China; b Xinjiang Key Lab of Conservation and Utilization of Plant Gene Resources, Chinese Academy of Sciences, Urumqi, China; c The Specimen Museum of Xinjiang Institute of Ecology and Geography, Chinese Academy of Sciences, Urumqi, China

**Keywords:** Chloroplast genome, Illumina sequencing, phylogenetic analysis, *Tamarix* L

## Abstract

*Tamarix* L. is of great ecological and economic significance in arid desert ecosystems. This study reports the complete chloroplast (cp) genomic sequences of *T. arceuthoides* Bunge and *T. ramosissima* Ledeb., which are currently unknown, by high-throughput sequencing. The cp genomes of *T. arceuthoides* 1852 and *T. ramosissima* 1829 were 156,198 and 156,172 bp in length, respectively, and contained a small single-copy region (SSC: 18,247 bp), a large single-copy region (LSC: 84,795 and 84,890 bp, respectively), and a pair of inverted repeat regions (IRs: 26,565 and 26,470 bp, respectively). The two cp genomes possessed 123 genes arranged in the same order, including 79 protein-coding, 36 tRNA, and eight rRNA genes. Of these, 11 protein-coding genes and seven tRNA genes contained at least one intron. The present study found that *Tamarix* and *Myricaria* are sister groups with the closest genetic relationship. The obtained knowledge could provide useful information for future phylogenetic, taxonomic, and evolutionary studies on Tamaricaceae.

## Introduction

*Tamarix* L. contains approximately 100 species that are primarily distributed in the arid and semi-arid areas of continental Asia and North Africa, along with intermittent distribution along the west coast of South Africa and parts of Europe (Zhang and Zhang 1990). There are 20 *Tamarix* species found in China, of which 16 species are known to propagate in Xinjiang (Liu 2014). Certain *Tamarix* species have been employed in ecological restoration projects to achieve objectives, including wind prevention, sand fixation, soil and water conservation, and climate regulation, showing great ecological and economic value in the maintenance of arid desert ecosystems (Baum 1978; Liu 2014). However, due to their similar morphological characteristics, the identification among this genus is frequently mistaken, which affects the effective development and utilization of some species in the genus *Tamarix*.

**Figure 1.**
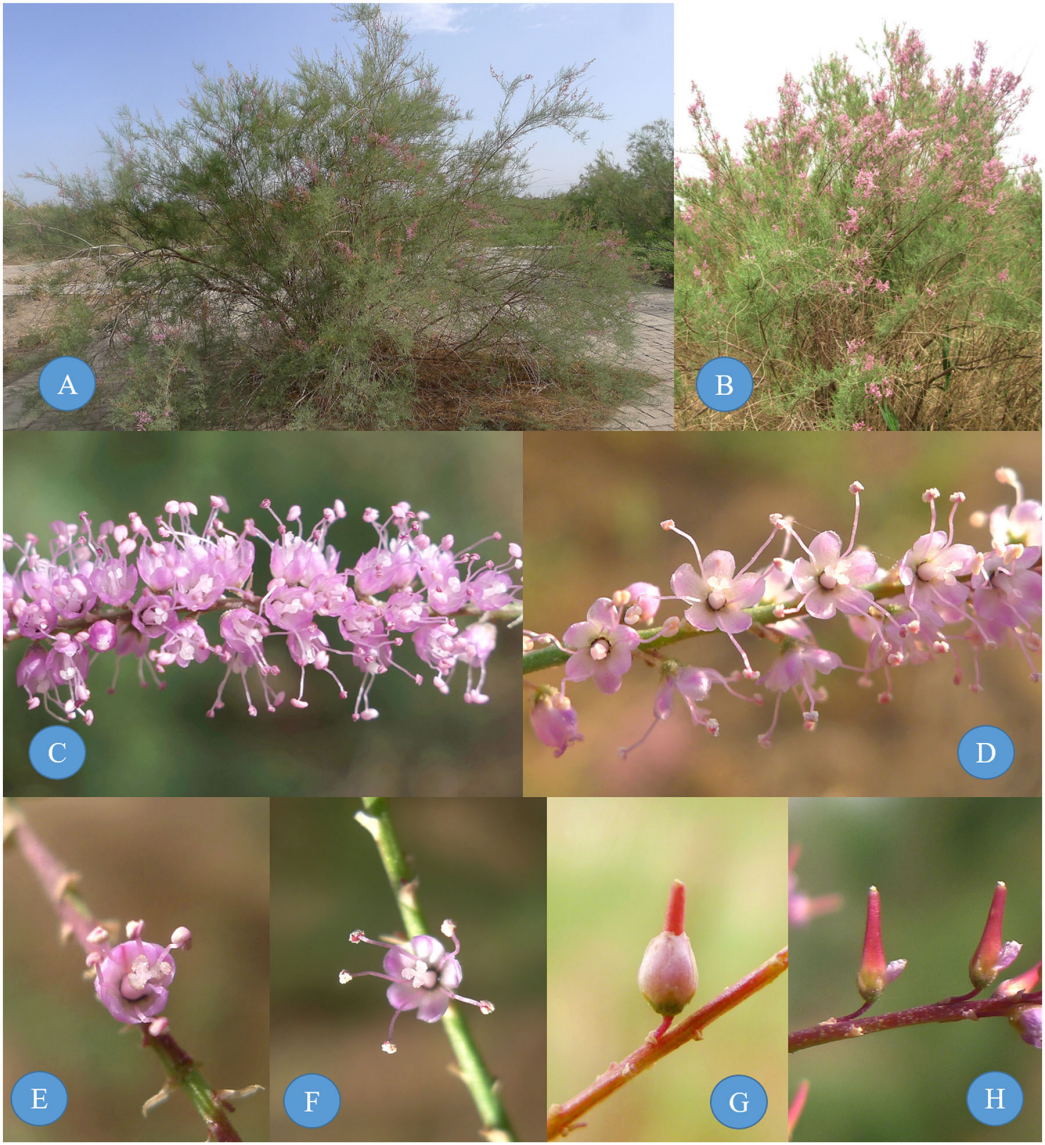
The individual, raceme, flower and capsule photograph of *T. arceuthoides* (B, D, F, and H) and *T. ramosissima* (A, C, E, and G), photo by Xiyong Wang at Turpan Eremophytes Botanical Garden. Main identifying traits: When *T. ramosissima* flowers, the petals are completely open, and the petals do not persist at fruit stage, but fall off directly and *T. arceuthoides* flowers cup shaped when flowering, petals persistent in fruit, attached to the base of the fruit.

The complete chloroplast (cp) genomes present an effective means of improving the rate of species identification and has been developed as a tool for plant phylogenetic studies at different taxonomic levels (Zhou et al. 2021; Wang et al. 2022). Till date, three *Tamarix* cp genomes, including those from two species and one unverified sequence (MN726883) have been published (Pang et al. 2021; Wang et al. 2021). This paucity of data has severely limited relevant research on this genus *Tamarix*. Here, we sequenced the cp genomes of *T. arceuthoides* Bunge 1852 and *T. ramosissima* Ledeb. 1829 (Figure 1), to conduct comprehensive research on the *Tamarix* cp genome, and serve as a reference for subsequent phylogenomic studies of the *Tamarix*.

## Materials

Samples of *T. arceuthoides* and *T. ramosissima* were collected from the Turpan Eremophytes Botanical Garden, Chinese Academy of Sciences, Turpan, Xinjiang, China (latitude 89°19′27.78″, longitude 42°85′71.92″ and latitude 89°11′22.56″, longitude 42°51′12.96″, respectively), and voucher specimens were deposited at the Specimen Museum of Xinjiang Institute of Ecology and Geography, Chinese Academy of Sciences (XJBI, Li Wenjun, liwenjunao@ms.xjb.ac.cn) with collection number LWJ-W-4, LWJ-W-7 and identifier Xi. Y. Wang. All materials utilized in the present study are listed in Supplementary Table.

## Methods

Leaf samples were dried in silica gel and stored at −20 °C for DNA extraction, which was performed using a plant genome extraction kit (DP320) obtained from Tiangen Biochemical Technology (Beijing, China) as per the manufacturer’s instructions. Extracted DNA was sequenced with 2 × 150 bp paired-end reads on the Illumina HiSeq X Ten platform at the Molecular Biology Experiment Center, Germplasm Bank of Wild Species in Southwest China.

Paired-end reads were assembled using GetOrganelle v. 1.7.1 (Jin et al. 2020). A complete circular assembly graph was checked and further extracted using Bandage version 0.8.1 (Wick et al. 2015). The genomes were automatically annotated using CpGAVAS (Liu et al. 2012) and PGA (https://github.com/quxiaojian/PGA), prior to manual adjustments using Geneious version 9.1.7 (Kearse et al. 2012), with the cp genome of *T. taklamakanensis* (MW125612) as a reference. The sequence data of *T. arceuthoides* and *T. ramosissima* are publicly available in the GenBank (https://www.ncbi.nlm.nih.gov/) under accession numbers ON620259 and ON620260. Organellar Genome Draw (OGDRAW) (Lohse et al. 2007) was used to illustrate the circular genome map.

To further explore the phylogenetic relationship of among *Tamarix* and its neighboring genus, maximum-likelihood (ML) analyses were conducted using RAxML-HPC v. 8 (Stamatakis 2014) with 1000 bootstrap replicates based on 11 cp genomes (after removing one inverted repeat (IR) region), including six *Tamarix* sequences, four *Myricaria* sequences, and *Reaumuria songarica* as outgroup (Supplementary Table). The evaluation of the most appropriate substitution models and the construction of the phylogenetic tree were both carried out on the CIPRES Science Gateway portal (Miller et al. 2011).

## Results

**Figure 2.**
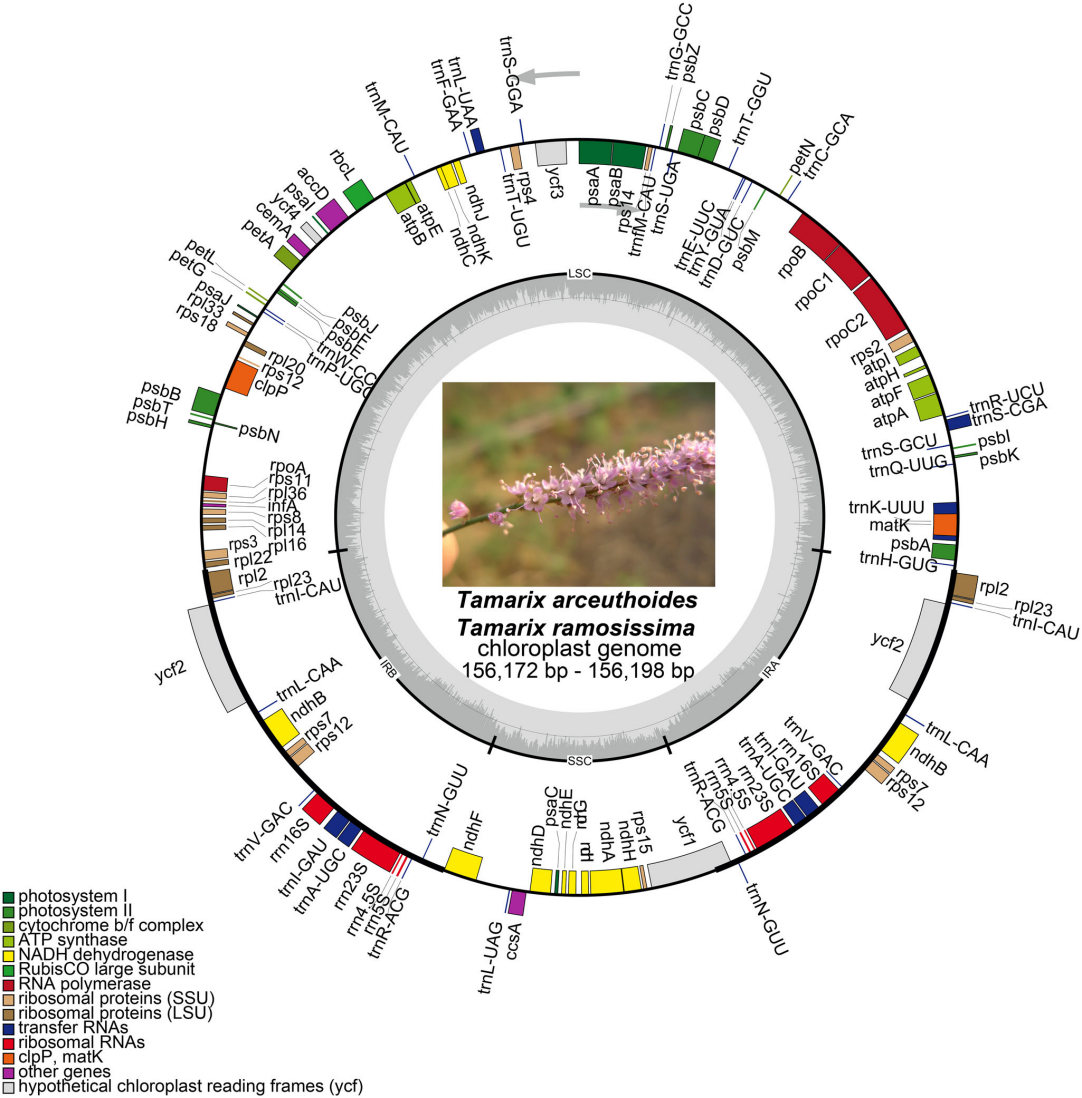
Chloroplast genome maps for *T. arceuthoides* and *T. ramosissima.* Genes on the inside of the circle are transcribed clockwise and those on the outside are transcribed counterclockwise. The darker gray inner circle corresponds to the GC content, whereas the lighter gray indicates the AT content. Different colors represent different functional genes. The thick lines of the large circle indicate the extent of the inverted repeat regions (IRa and IRb) that separate the genome into small single-copy (SSC) and large single-copy (LSC) regions, respectively.

The new cp genomic sequences were found to occupy a circular confirmation, 156,172 and 156,198 bp in length, respectively, comprising a small single-copy region (SSC: 18,247 bp) as well as a large single-copy region (LSC: 84,795 and 84,890 bp, respectively) that were separated by a pair of inverted repeat regions (IRs: 26,565 and 26,470 bp, respectively). Both the cp genomes possessed 123 genes that were arranged in the same order, including 79 protein-coding genes, 36 tRNA genes, and eight rRNA genes. Of these, 11 protein-coding and seven tRNA genes contained at least one intron (Figure 2). These two sequences had 36.4% and 36.5% GC content, respectively. Comparison of the two cp genomes to previously published data revealed a high level of gene synteny with one publicly available genome data sets from *T. taklamakanensis* (MW125612).

**Figure 3.**
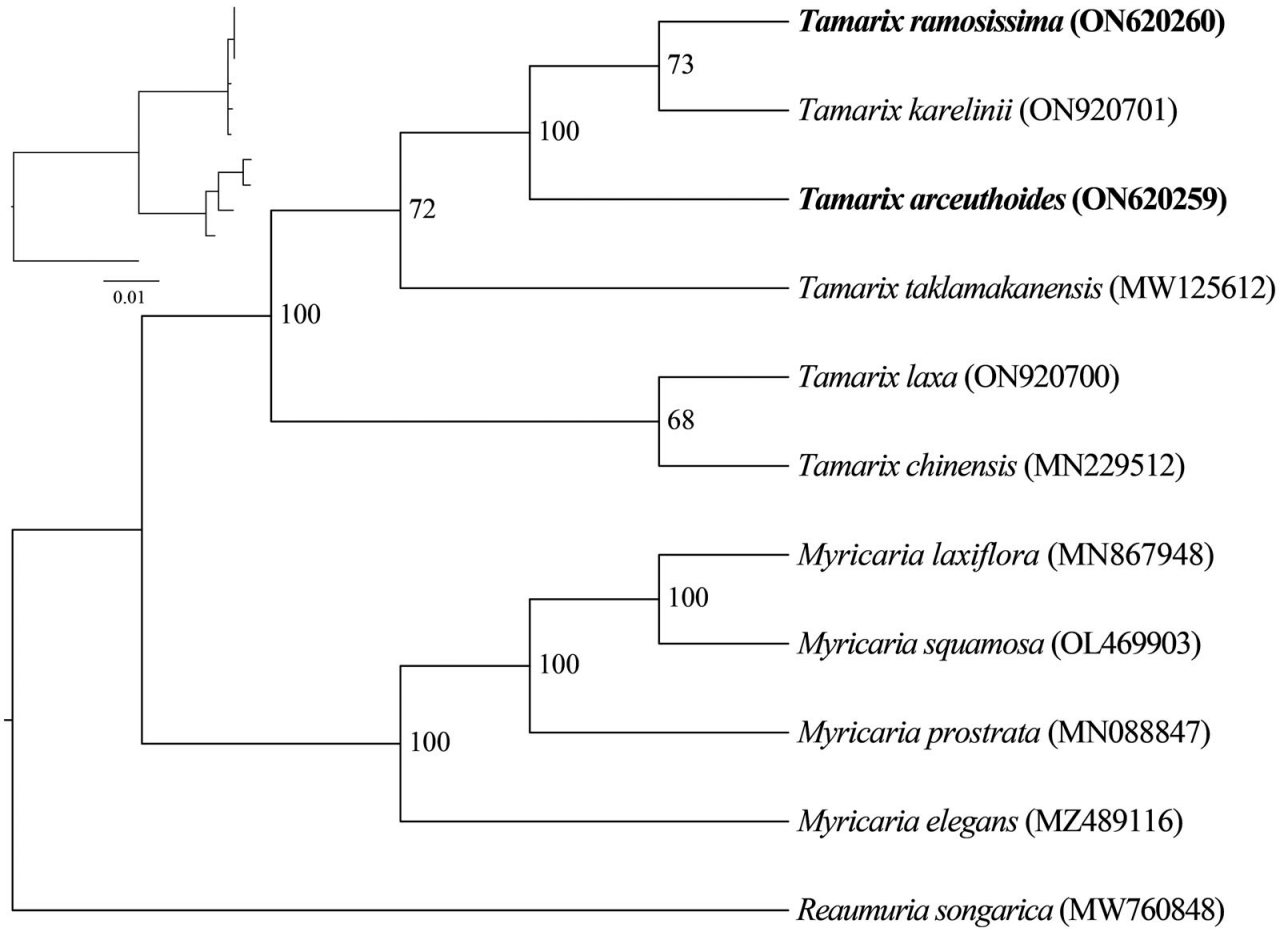
Phylogenetic trees inferred from maximum-likelihood (ML) analyses based on 12 complete chloroplast genomes using *Reaumuria songarica* as an outgroup with 1000 bootstraps replicates. The numbers above the branches indicate the bootstrap values. The following sequences were used: *Reaumuria songarica* MW760848 (Duan Y et al. 2021), *Myricaria elegans* MZ489116 (Su T and Han M 2021), *Myricaria laxiflora* MN867948 (Wang et al. 2020), *Myricaria prostrata* MN088847 (Chi X 2020), *Myricaria squamosa* OL469903 (Yu L 2022), *Tamarix chinensis* MN229512 (Chi 2019), *Tamarix karelinii* ON920701 (Song S 2022), *Tamarix laxa* ON920700 (Song S 2022), and *Tamarix taklamakanensis* MW125612 (Yang T 2020).

The phylogenetic tree analysis revealed that *Tamarix* and *Myricaria* were sister groups (BS = 100%). *Tamarix* was divided into three main lineages, the new sequences *T. arceuthoides* and *T. ramosissima* clustered with *T. karelinii* together (Figure 3).

## Discussion and conclusions

We report the cp genomes of *T. arceuthoides* and *T. ramosissima*. The structures obtained for the two cp genomes in this study are consistent with previous findings (Pang 2021). Our study demonstrates that plastome studies can provide useful information for future phylogenetic, taxonomic, and evolutionary studies on Tamaricaceae. However, the complete cp genomes are not distinct between the *T. arceuthoides* and *T. ramosissima*, indicating that the molecular identification of the genus *Tamarix* might require to select highly variable regions in cp genomes, or add additional nrDNA sequences.

## Supplementary Material

Supplemental Material

Supplemental Material

Supplemental Material

Supplemental Material

Supplemental Material

Supplemental Material

## Data Availability

The genome sequence data that support the findings of this study are openly available in GenBank of NCBI at https://www.ncbi.nlm.nih.gov under accession nos. ON620259 and ON620260. The associated BioProjects are PRJNA890384 and PRJNA890011; SRA are SRR21901739 and SRR21889044; and the Bio-Sample numbers are SAMN31264515 and SAMN31266535, respectively.
