# Supplementary figures and images for: The complete chloroplast genome sequence of Tamarix arceuthoides Bunge and Tamarix ramosissima Ledeb. (Tamaricaceae)
Source: Mitochondrial DNA B Resour. 2023 May 11;8(5):541–5. doi: 10.1080/23802359.2023.2209215 (PMC10177690; doi:10.1080/23802359.2023.2209215)

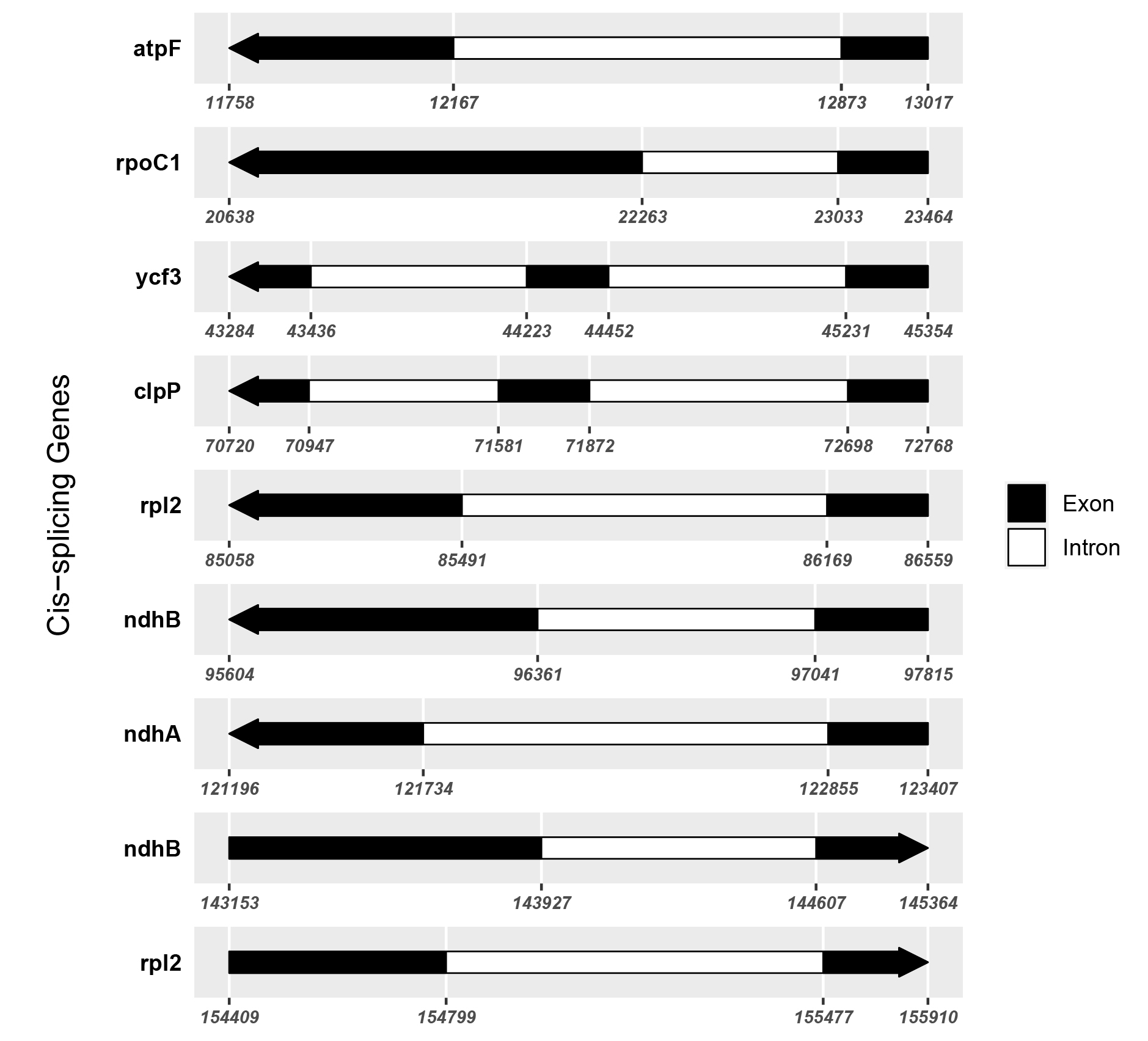

Supplement: Supplemental Material [file TMDN_A_2209215_SM0704.jpg]

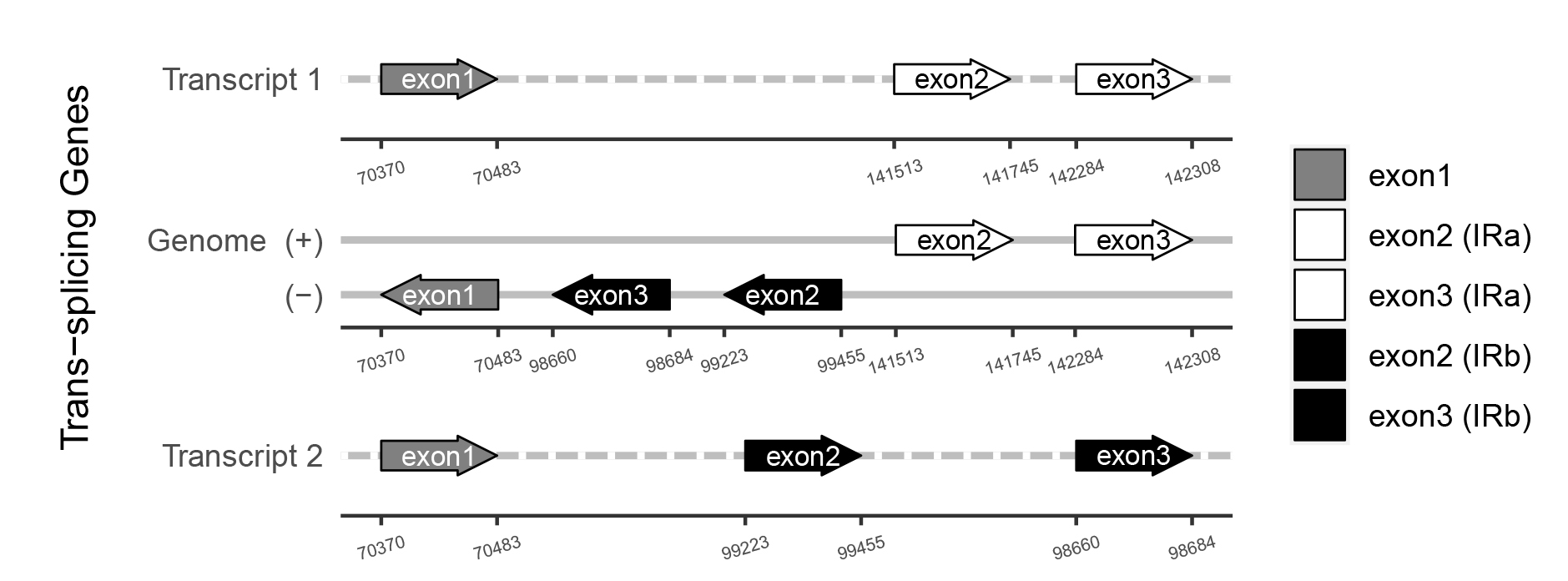

Supplement: Supplemental Material [file TMDN_A_2209215_SM0701.jpg]

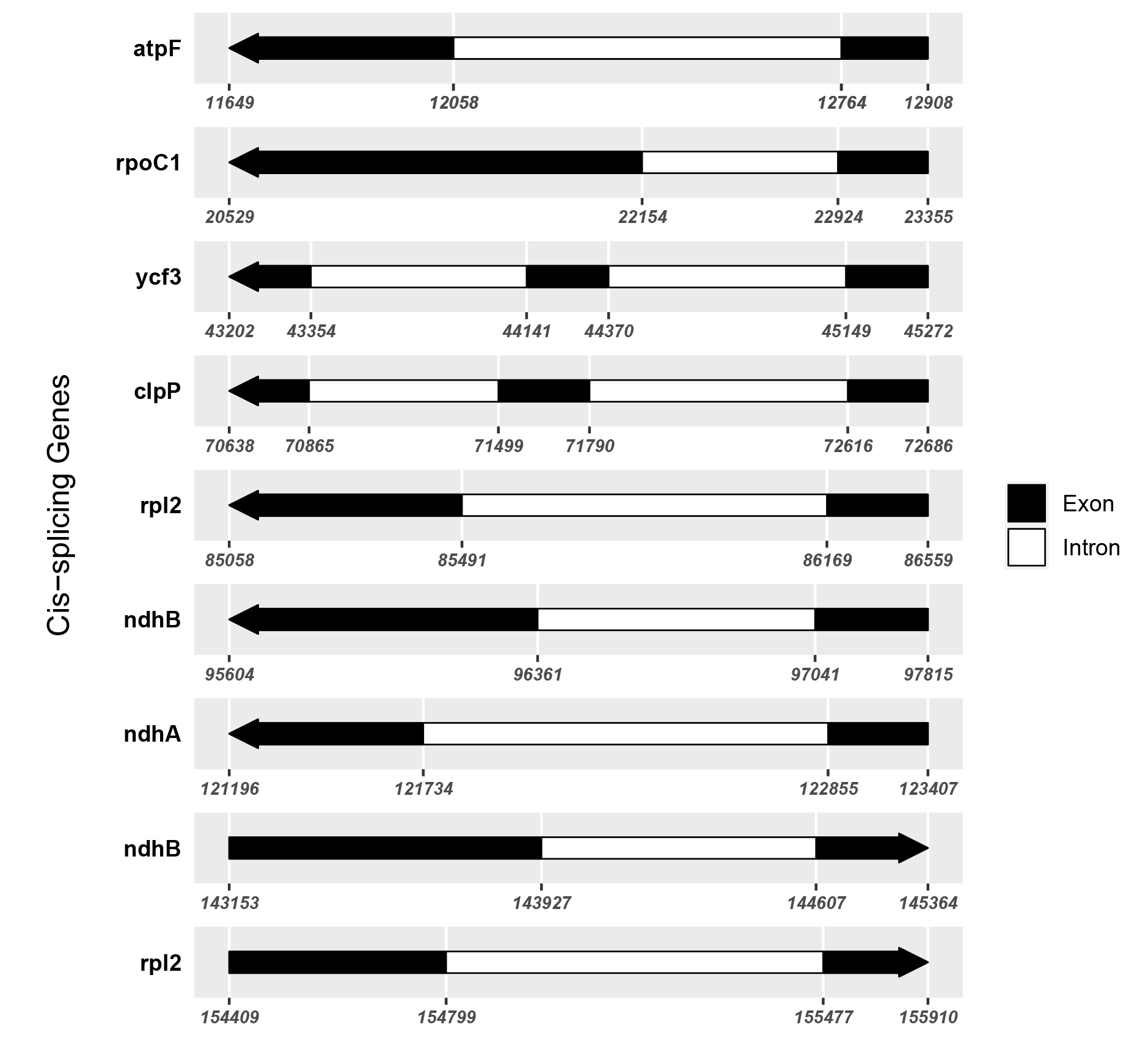

Supplement: Supplemental Material [file TMDN_A_2209215_SM0694.jpg]

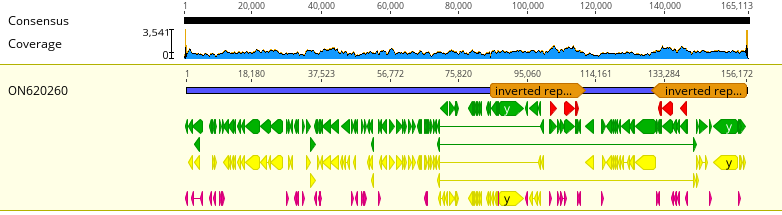

Supplement: Supplemental Material [file TMDN_A_2209215_SM0686.png]

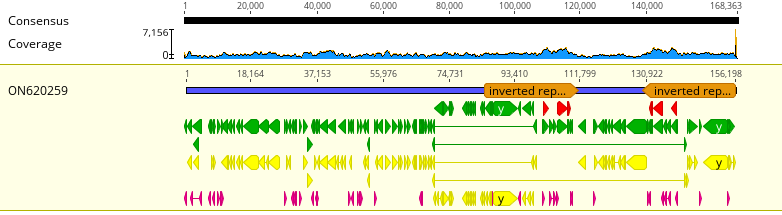

Supplement: Supplemental Material [file TMDN_A_2209215_SM0685.png]
