# Supplementary material for: The complete chloroplast genome sequence of Tamarix arceuthoides Bunge and Tamarix ramosissima Ledeb. (Tamaricaceae)
Source: Mitochondrial DNA B Resour. 2023 May 11;8(5):541–5. doi: 10.1080/23802359.2023.2209215 (PMC10177690; doi:10.1080/23802359.2023.2209215)
Supplement: Supplemental Material [file TMDN_A_2209215_SM0682.docx]

Supplementary Table. Materials utilized in the study

| Sample ID | Species | collection | Voucher specimens | Identifier | Status | Accession number |
| --- | --- | --- | --- | --- | --- | --- |
| 1 | *Tamarix arceuthoides* | LWJ-W-7 | tlf2020010 | Xi. Y. Wang | Fresh leaves | ON620259 |
| 2 | *Tamarix ramosissima* | LWJ-W-4 | tlf2020012 | Xi. Y. Wang | Fresh leaves | ON620260 |
| 3 | *Tamarix chinensis* | NCBI |  |  |  | MN229512 |
| 4 | *Tamarix taklamakanensis* | NCBI |  |  |  | MW125612 |
| 5 | *Tamarix laxa* | NCBI |  |  |  | ON920700 |
| 6 | *Tamarix karelinii* | NCBI |  |  |  | ON920701 |
| 7 | *Reaumuria songarica* | NCBI |  |  |  | MW760848 |
| 8 | *Myricaria squamosa* | NCBI |  |  |  | OL469903 |
| 9 | *Myricaria prostrata* | NCBI |  |  |  | MN088847 |
| 10 | *Myricaria laxiflora* | NCBI |  |  |  | MN867948 |
| 11 | *Myricaria elegans* | NCBI |  |  |  | MZ489116 |
